# The MOG antibody non-P42 epitope is predictive of a relapsing course in MOG antibody-associated disease

**DOI:** 10.1136/jnnp-2023-332851

**Published:** 2024-01-30

**Authors:** Ganesha Liyanage, Benjamin P Trewin, Joseph A Lopez, Jane Andersen, Fiona Tea, Vera Merheb, Kristy Nguyen, Fiona X Z Lee, Marzena J Fabis-Pedrini, Alicia Zou, Ali Buckland, Anthony Fok, Michael H Barnett, Stephen W Reddel, Romain Marignier, Aseel El Hajj, Mastura Monif, Anneke van der Walt, Jeannette Lechner-Scott, Allan G Kermode, Tomas Kalincik, Simon A Broadley, Russell C Dale, Sudarshini Ramanathan, Fabienne Brilot

**Affiliations:** 1 Brain Autoimmunity Group, Kids Neuroscience Centre, Kids Research at the Children’s Hospital at Westmead, Sydney, New South Wales, Australia; 2 School of Medical Sciences, Faculty of Medicine and Health, The University of Sydney, Sydney, New South Wales, Australia; 3 Translational Neuroimmunology Group, Kids Neuroscience Centre, Kids Research at the Children’s Hospital at Westmead, Sydney, New South Wales, Australia; 4 Sydney Medical School, Faculty of Medicine and Health, The University of Sydney, Sydney, New South Wales, Australia; 5 Centre for Neuromuscular and Neurological Disorders, Perron Institute for Neurological and Translational Science, The University of Western Australia, Sir Charles Gairdner Hospital, QEII Medical Centre, Nedlands, Western Australia, Australia; 6 Centre for Molecular Medicine and Innovative Therapeutics, Murdoch University, Murdoch, Western Australia, Australia; 7 Department of Neurology, Monash Health, Clayton, Victoria, Australia; 8 Brain and Mind Centre, The University of Sydney, Camperdown, New South Wales, Australia; 9 Department of Neurology, Concord Repatriation General Hospital, Sydney, New South Wales, Australia; 10 Service de Neurologie, Sclérose en Plaques, Pathologies de la Myéline et Neuro Inflammation, and Centre de Référence des Maladies Inflammatoires Rares du Cerveau et de la Moelle, Hôpital Neurologique Pierre Wertheimer and Centre des Neurosciences de Lyon, INSERM 1028 et CNRS UMR5292, Lyon, France; 11 Université Claude Bernard Lyon 1, Lyon, France; 12 Multiple Sclerosis and Neuroimmunology Research Groups, Department of Neuroscience, Monash University, Clayton, Victoria, Australia; 13 Department of Neurology, John Hunter Hospital, Newcastle, New South Wales, Australia; 14 Hunter Medical Research Institute, The University of Newcastle, New Lambton Heights, New South Wales, Australia; 15 School of Medicine and Public Health, The University of Newcastle, Callaghan, New South Wales, Australia; 16 Institute for Immunology and Infectious Diseases, Murdoch University, Perth, Western Australia, Australia; 17 Clinical Outcomes Research Unit (CORe), Department of Medicine, The University of Melbourne, Melbourne, Victoria, Australia; 18 Neuroimmunology Centre, Department of Neurology, The Royal Melbourne Hospital, Melbourne, Victoria, Australia; 19 School of Medicine and Dentistry, Griffith University, Gold Coast, Queensland, Australia; 20 Department of Neurology, Gold Coast University Hospital, Southport, Queensland, Australia; 21 Clinical Neuroimmunology Group, Kids Neuroscience Centre, Kids Research at the Children’s Hospital at Westmead, Sydney, New South Wales, Australia

**Keywords:** NEUROIMMUNOLOGY, IMMUNOLOGY, MULTIPLE SCLEROSIS

## Abstract

**Background:**

Myelin oligodendrocyte glycoprotein (MOG) IgG seropositivity is a prerequisite for MOG antibody-associated disease (MOGAD) diagnosis. While a significant proportion of patients experience a relapsing disease, there is currently no biomarker predictive of disease course. We aim to determine whether MOG-IgG epitopes can predict a relapsing course in MOGAD patients.

**Methods:**

MOG-IgG-seropositive confirmed adult MOGAD patients were included (n=202). Serum MOG-IgG and epitope binding were determined by validated flow cytometry live cell-based assays. Associations between epitopes, disease course, clinical phenotype, Expanded Disability Status Scale and Visual Functional System Score at onset and last review were evaluated.

**Results:**

Of 202 MOGAD patients, 150 (74%) patients had MOG-IgG that recognised the immunodominant proline42 (P42) epitope and 115 (57%) recognised histidine103/serine104 (H103/S104). Fifty-two (26%) patients had non-P42 MOG-IgG and showed an increased risk of a relapsing course (HR 1.7; 95% CI 1.15 to 2.60, p=0.009). Relapse-freedom was shorter in patients with non-P42 MOG-IgG (p=0.0079). Non-P42 MOG-IgG epitope status remained unchanged from onset throughout the disease course and was a strong predictor of a relapsing course in patients with unilateral optic neuritis (HR 2.7, 95% CI 1.06 to 6.98, p=0.038), with high specificity (95%, 95% CI 77% to 100%) and positive predictive value (85%, 95% CI 45% to 98%).

**Conclusions:**

Non-P42 MOG-IgG predicts a relapsing course in a significant subgroup of MOGAD patients. Patients with unilateral optic neuritis, the most frequent MOGAD phenotype, can reliably be tested at onset, regardless of age and sex. Early detection and specialised management in these patients could minimise disability and improve long-term outcomes.

**WHAT IS ALREADY KNOWN ON THIS TOPIC**

- Myelin oligodendrocyte glycoprotein (MOG) IgG seropositivity is a prerequisite in the diagnosis of MOG antibody-associated disease (MOGAD). While a significant proportion of patients are affected by a relapsing disease, there is currently no biomarker predictive of disease course.

**WHAT THIS STUDY ADDS**

- In this retrospective cohort study, a subgroup of adult MOGAD patients with MOG-IgG bound to a non-dominant MOG epitope and showed a significantly increased risk of a relapsing course.

**HOW THIS STUDY MIGHT AFFECT RESEARCH, PRACTICE OR POLICY**

- These findings suggest that non-P42 MOG-IgG may be the first diagnostic predictor of a relapsing course in a distinct subgroup of MOGAD patients.

## Introduction

Myelin oligodendrocyte glycoprotein antibody-associated disease (MOGAD) is a central nervous system (CNS) disease characterised by the presence of IgG autoantibodies targeting MOG (MOG-IgG) and demyelinating lesions affecting the optic nerve, spinal cord, brain or brainstem in children and adults.^1–9^ While some patients experience a monophasic disease course, approximately 40% of adults and 30% of children experience a relapse within 5 years of disease onset^10–12^ and early evidence suggests that longer follow-up may reveal higher rates of relapse.^13^ Higher degree of disability may be associated with recurrent demyelinating episodes.^11 14 15^ Thus, a key priority in the prognostication of MOGAD is identifying patients who are at risk of relapse as early as possible, such as at disease onset. Such prediction could aid management by selecting appropriate immunotherapy while avoiding unnecessary immunosuppression in monophasic patients. Furthermore, the early identification and inclusion of patients at risk of relapse in clinical trials would increase the statistical power of studies aimed at discerning effective therapeutic strategies for relapsing MOGAD.^2^

Several factors associated with relapse have been investigated. One of these is persisting MOG-IgG seropositivity throughout the disease.^11 16–20^ Furthermore, optic neuritis (ON), younger age in adults or early relapse within the first 12 months of onset has shown to be associated with an elevated risk of relapsing disease.^21 22^ However, a true predictor of a relapsing course, assessable prior to clinical relapse, is still lacking.

The detection of MOG-IgG by cell-based assays is an essential criterion in MOGAD diagnosis and can be used to determine the seropositivity^2 23–25^ and epitope^26–28^ of MOG-IgG. MOG-IgG has been reported to bind to amino acids at two key antigenic binding regions, or epitopes for brevity, located on the extracellular Ig-like domain of MOG: proline42 (P42),^27 28^ and histidine103/serine104 (H103/S104).^28 29^ Previously, we reported that 75% of adult patients, whose MOG-IgG bound an amino acid other than P42, exhibited a relapsing course.^27^ However, whether this preliminary finding had any diagnostic and prognostic value in predicting relapsing course for clinical purposes was not determined.

Here, we have undertaken an in-depth analysis of MOG-IgG binding patterns and their associations with relapse in a large cohort of adult MOGAD patients with detailed clinical phenotypes. We hypothesised that non-P42 epitope binding would be predictive of a relapsing course.

## Methods

### Study cohort

Adult patients who were MOG-IgG-seropositive with a clinical phenotype consistent with MOGAD (n=202, >16 years of age at disease onset) were recruited between January 2013 and June 2022 out of n=345 MOGAD adults tested seropositive (minimum follow-up time of 1 year; median follow-up 4 years, IQR 2.2–7.2) (table 1). Patients were diagnosed in accordance with the recently published International MOGAD criteria.^2^ Clinical episodes were reported by neurologists and the time of collection of serum samples were classified as onset, remission or relapse. A relapse was defined as new CNS symptoms or signs lasting >24 hours, in the absence of other causes, and clinically and/or radiologically compatible with a MOGAD episode (table 1).^2^ Remission was defined as disease stability >30 days after an onset or relapse. Clinical data were collected where available and included episode phenotype, date of onset episode and last clinical review by a neurologist, Expanded Disability Status Scale (EDSS) and Visual Functional System Score (VFSS) scores at nadir of the onset episode and at last review, and date of first relapse (table 1). MOGAD phenotypes were categorised at onset as either ON, which was subgrouped into unilateral ON (UON) or bilateral ON (BON), or ON not otherwise specified, transverse myelitis (TM), ON and TM, brain (including brainstem or cerebellar deficits and acute disseminated encephalomyelitis), and ‘mixed’ if patients experienced a combination of any phenotypes other than ON and TM (table 1).

**Table 1:** Demographics, clinical and MOG-IgG characteristics of MOGAD patients

	Total	Monophasic	Relapsing	P value*
n	202	65	137	
Age, median, year (IQR)	39.89 (30.85–51.24)	37.96 (28.43–54.22)	40.53 (31.60–50.75)	0.681
Sex, male, n (%)	81 (40.1)	27 (41.5)	54 (39.4)	0.893
Follow-up time, median, year (IQR)	4.07 (2.18–7.23)	2.79 (1.65–4.21)	5.33 (2.47–9.60)	<0.001
Time to first relapse, median, month (IQR), n†	–	–	7.03 (3.00–24.63), 113	–
ARR, median (IQR), n†	–	–	0.38 (0.19–0.57), 86	–
Phenotype at onset, n (%)				0.42
UON	76 (37.6)	22 (33.8)	54 (39.4)	
BON	61 (30.2)	22 (33.8)	39 (28.5)	
ON (not otherwise specified)	5 (2.5)	0 (0.0)	5 (3.6)	
ON/TM	7 (3.5)	4 (6.2)	3 (2.2)	
TM	35 (17.3)	10 (15.4)	25 (18.2)	
Brain	14 (6.9)	5 (7.7)	9 (6.6)	
Mixed	4 (2.0)	2 (3.1)	2 (1.5)	
Phenotype over whole disease course, n (%)				–
UON‡	62 (30.7)	22 (33.8)	40 (29.2)	
BON	31 (15.3)	22 (33.8)	9 (6.6)	
ON (not otherwise specified)	2 (1.0)	0 (0.0)	2 (1.5)	
ON (mixed)§	24 (11.9)	0 (0.0)	24 (17.5)	
ON/TM	23 (11.4)	4 (6.2)	19 (13.9)	
TM¶	25 (12.4)	10 (15.4)	15 (10.9)	
Brain	9 (4.5)	5 (7.7)	4 (2.9)	
Mixed	26 (12.9)	2 (3.1)	24 (17.5)	
Disease stage at first serum collection, n (%)				–
Onset	80 (39.6)	39 (60.0)	41 (29.9)	
Relapse	32 (15.8)	–	32 (23.4)	
Remission	73 (36.1)	25 (38.5)	48 (35.0)	
Remission before first relapse	15 (7.4)	–	15 (10.9)	
Unknown	2 (1.0)	1 (1.5)	1 (0.7)	
EDSS, median (IQR)				
Onset**, n†	3.00 (2.00–4.00), 122	3.00 (2.00–4.00), 44	3.00 (2.00–4.00), 78	0.628
Last Review, n†	1.00 (0.00–2.00), 148	0.00 (0.00–1.00), 44	1.50 (0.00–3.00), 104	<0.001
VFSS, median (IQR)				
Onset**, n†	3.00 (0.00–5.00), 111	2.00 (0.00–5.00), 40	3.00 (0.50–5.00), 71	0.537
Last review, n†	0.00 (0.00–2.00), 134	0.00 (0.00–0.25), 40	1.00 (0.00–2.00), 94	0.001
MOG-IgG titre, clear positive, n (%)††	77 (96.2)	38 (97.4)	39 (95.1)	0.084
Non-P42 MOG-IgG, n (%)	52 (25.7)	8 (12.3)	44 (32.1)	0.003
Non-H103/S104 MOG-IgG, n (%)	87 (43.1)	28 (43.1)	59 (43.1)	1
Combined P42 and H103/S104 epitope, n (%)				0.013
P42 and H103/S104	80 (39.6)	30 (46.2)	50 (36.5)	
P42 and non-H103/S104	70 (34.7)	27 (41.5)	43 (31.4)	
H103/S104 and non-P42	35 (17.3)	7 (10.8)	28 (20.4)	
Non-P42 and non-H103/S104	17 (8.4)	1 (1.5)	16 (11.7)	

*P values were computed based on comparisons between monophasic and relapsing patients.

†Number of patients with ON, TM, brain and Mixed phenotypes for whom the data were available.

‡UON as a phenotype at relapse was 4.5 times more frequent than BON.

§ON (mixed) refers to subsequent attacks of UON and BON.

¶Included short TM (n=7), longitudinal extended TM (n=4), a combination of short TM and longitudinal extended TM (n=4) and TM not otherwise specified (n=10).

**EDSS and VFSS scores assessed at nadir of the onset.

††MOG-IgG titre was calculated for patient sera collected at onset.

AAR, annualised rate of relapse; BON, bilateral optic neuritis; EDSS, Expanded Disability Status Scale; MOG, myelin oligodendrocyte glycoprotein; ON, optic neuritis; TM, transverse myelitis; UON, unilateral optic neuritis; VFSS, Visual Functional Systems Score.

### MOG-IgG epitope testing

MOG-IgG testing (IgGs (H+L) and IgG1) was performed on patient sera with a validated flow cytometry live cell-based assay (online supplemental methods) and as previously described.^23 27^ The MOG-IgG test has been available in Australia since December 2012. Two antigenic binding regions including P42 or H103/S104, herein referred to as P42 and H103/S104 MOG-IgG epitopes for brevity, were assessed using two MOG mutants: MOG P42S and MOG H103A/S104E. The MOG P42S mutant contained full-length human MOG with a mutation at position 42, where proline was substituted for serine.^27 28^ The MOG H103A/S104E mutant consisted of full-length human MOG in which the histidine and serine at positions 103 and 104 were substituted with alanine and glutamic acid.^28^ While some structural changes may be induced by the mutations at residues 42, 103 and 104, none of them significantly altered the high expression of surface MOG P42S or MOG H103A/S104E mutants compared with human wild-type (online supplemental methods).^27 28^ Binding to P42 and H103/S104 epitopes were determined by subtracting the binding to MOG P42S or MOG H103A/S104E mutants from human MOG. Optimal thresholds for P42 and H103/S104 MOG-IgG binding were calculated after receiver-operating-characteristic (ROC) analysis (online supplemental figure 1A-D).^30^ Epitope and non-epitope groups were plotted such that MOG-IgG binding to P42 and H103/S104 were above the threshold while MOG-IgG binding to non-P42 and non-H103/S104 were below and appeared negative.

10.1136/jnnp-2023-332851.supp1 Supplementary data

### Statistical analysis

Pairwise comparisons between continuous data were made using the Wilcoxon rank-sum test, with Bonferroni correction for multiple pairwise tests. Fisher’s exact test was used to compare nominal variables and association of epitopes groups and acute immunotherapy. Univariate and multivariable Cox proportional hazard models were performed to test for associations between epitopes, disease course and clinical phenotypes. Kaplan-Meier analysis was performed and included relapsing and monophasic patients, with monophasic patients included as censored events. A number of censored observations are shown in figures. P values were two sided, and statistical significance was set at p<0.05. All statistical analyses were performed by using R V.4.0.5 (The R Foundation).

## Results

### P42 and H103/S104 were key epitopes targeted by MOG-IgG in adult MOGAD patients

**Figure 1:**
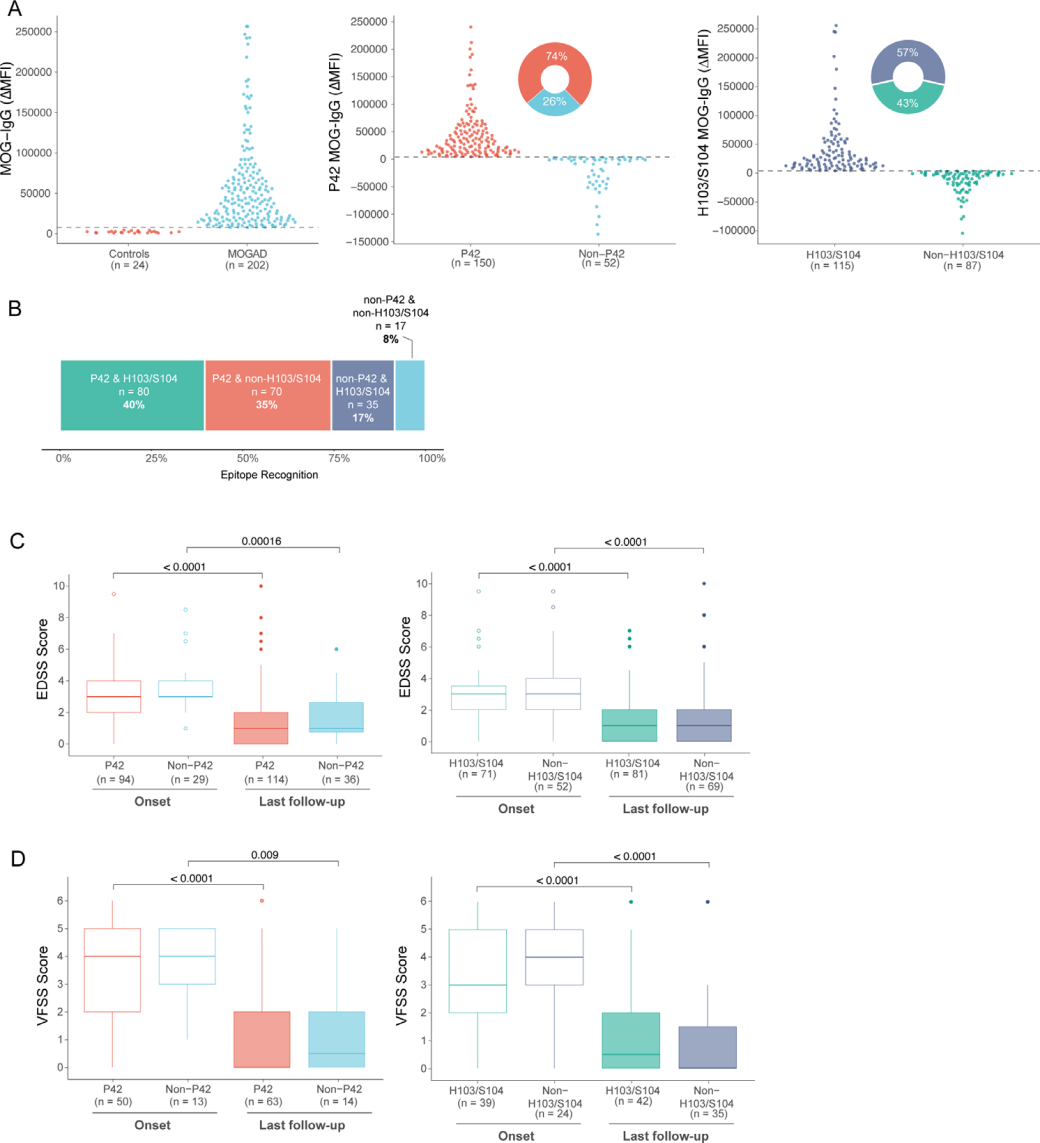
P42 and H103/S104 epitopes were the key MOG-IgG epitopes in adults. (A) MOG-IgG was assessed in 202 patient sera, and P42 and H103/S104 epitope statuses were determined. (B) Sera with MOG-IgG recognise one, both or neither of the two immunodominant epitopes. (C) EDSS and (D) VFSS scores were compared between MOG-IgG epitopes, and between disease onset and last follow-up date. EDSS, Expanded Disability Status Scale; MOG, myelin oligodendrocyte glycoprotein; VFSS, Visual Functional Systems Score.

The MOG-IgG epitope serum profile was characterised in a total of 202 adult MOG-IgG-seropositive confirmed MOGAD patients. The demographic and baseline clinical characteristics of this cohort are summarised in table 1 and are consistent with prior studies of adult MOGAD cases.^11 13 27 31^ Patients had a monophasic (65/202; 32%) or relapsing disease course (137/202; 68%). The duration of disease follow-up was longer in relapsing patients compared with monophasic patients (table 1). ON was the most frequent clinical phenotype at onset (table 1). Concurrent with previous studies,^27 28^ P42 was the most commonly recognised epitope (150/202; 74%), whereas 52 (26%) sera recognised an epitope outside of the P42 epitope (non-P42) (figure 1A). The H103/S104 residue was also found to be part of a key epitope recognised by 115 (57%) sera, whereas 87 (43%) did not bind to H103/S104 (figure 1A). Overall, MOG-IgG commonly recognised both P42 and H103/S104 epitopes (n=80, 40%) while a minority recognised a non-P42 and non-H103/S104 epitope (n=17, 8%) (figure 1B). MOG-IgG binding to P42, rather than the H103/S104 epitope, was prevalent in 70 sera (35%) while 35 (17%) recognised the H103/S104 but not the P42 epitope.

There was no significant difference in sex, age of patients and time between onset and first collected sample between epitope groups (online supplemental figure 2A,B). There was no significant difference in EDSS and VFSS scores between epitope groups, or combination thereof, at either onset or last review (figure 1C,D, online supplemental figure 3A,B). Patients displayed a lower EDSS and VFSS score at the last review compared with onset scores within all epitope categories, except in the non-P42 and non-H103/S104 epitope group in which there was no significant difference in VFSS scores at onset and last review, likely due to small sample size (figure 1C,D, online supplemental table 1).

### Patients with non-P42 MOG-IgG exhibited increased risk of a short-term relapsing course compared with patients with P42 MOG-IgG

**Figure 2:**
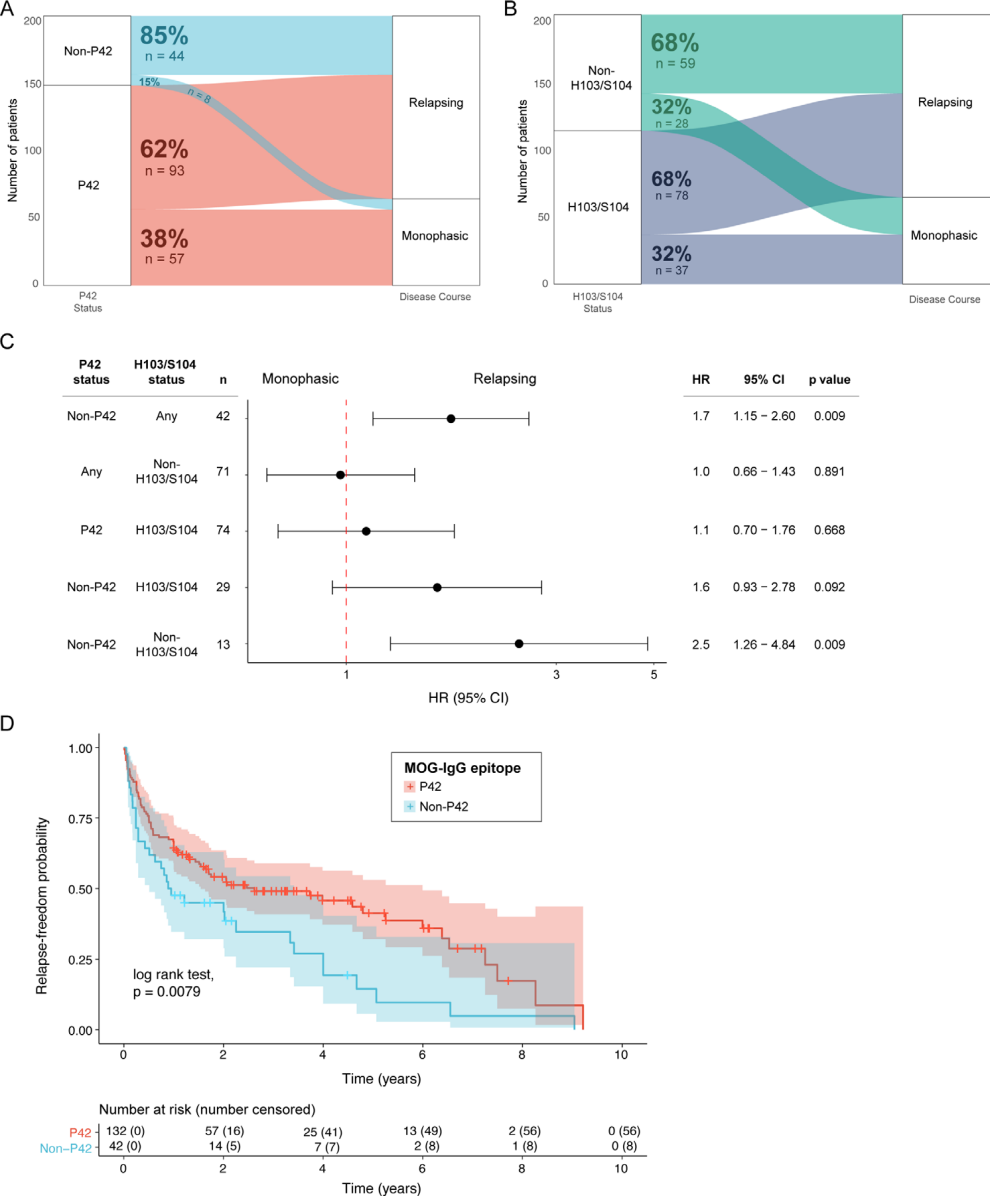
Non-P42 MOG-IgG was associated with a relapsing disease course. Alluvial plots demonstrating the proportion of MOGAD patients (n=202) with monophasic and relapsing disease course and their (A) P42 MOG-IgG and (B) H103/S104 MOG-IgG status. (C) Univariate Cox proportional hazard model illustrating the HR of a relapsing course in each epitope group compared with the corresponding reference group as follows: patients with MOG-IgG binding to a P42 epitope (top row), H103/S104 epitope (second row) and P42 and non-H103/S104 epitope (last three rows). (D) Kaplan-Meier curve showing the relapse-freedom period of patients with P42 MOG-IgG compared with non-P42 MOG-IgG (number of censored observations and number at risk are shown below the curve). 95% CIs are shown in shaded areas on both sides of the survival curves. MOGAD, myelin oligodendrocyte glycoprotein antibody-associated disease.

We next sought to examine whether epitopes could predict a relapsing disease course. Interestingly, 44/52 (85%) patients with non-P42 MOG-IgG had a relapsing course, compared with 93/150 (62%) patients with P42 MOG-IgG (figure 2A). This association remained true when patient sera were tested with an anti-human IgG1 secondary antibody, in which 41 of 45 patients with non-P42 MOG-IgG were confirmed. This ensured applicability to international MOG-IgG testing centres (online supplemental figure 1E,F). In contrast, there was no difference in the proportion of relapsing patients whose sera bound a H103/S104 and non-H103/S104 epitope (figure 2B). Univariate Cox proportional hazard model revealed that patients with non-P42 MOG-IgG had 70% of increased risk of a relapsing course compared with patients whose MOG-IgG bound P42 (HR 1.7; 95% CI 1.15 to 2.60; p=0.009; figure 2C), highlighting an association between a non-P42 epitope and a relapsing course. However, there was no change in the hazard of having a relapsing course based on H103/S104 epitope status (HR 1; 95% CI 0.66 to 1.43; p=0.891; figure 2C). MOG-IgG-seropositive patients whose sera recognised the H103/S104 epitope, irrespective of the binding to the P42 epitope, were not more likely to exhibit a relapsing course. However, patients whose MOG-IgG recognised a non-P42 and non-H103/S104 epitope had higher risk of exhibiting a relapsing course than those who recognised the combination of P42 and non-H103/S104 epitopes (HR 2.5; 95% CI 1.26 to 4.84; p=0.009). We next focused on the P42 epitope due to higher applicability of testing one rather than two epitopes in diagnostics. There was no difference in phenotype, disease severity or MOG-IgG titre at onset between relapsing patients with sera binding the P42 epitope compared with the non-P42 epitope (online supplemental table 2). The duration of relapse-freedom was significantly longer in patients with a P42 epitope than those with a non-P42 epitope (p=0.0079, log-rank test, median time to first relapse of 2.7 years, 95% CI 1.54 to 5.99 vs 0.9 years, 95% CI 0.51 to 3.41; figure 2D). For instance, at 5 years post-onset, the relapse-freedom probability of patients with non-P42 MOG-IgG epitope was 0.14 (95% CI 0.06 to 0.37), compared with 0.41 (95% CI 0.32 to 0.53), among patients with P42 MOG-IgG epitope. Notably, there was no significant association between acute corticosteroid immunotherapy during the first clinical episode or lack thereof, and the two epitope groups (p=0.159, online supplemental figure 4), suggesting that, although treatment could influence MOGAD disease course, patients with a specific MOG-IgG epitope were not treated differently during the first episode.

### The association between a non-P42 epitope and a relapsing disease course was the strongest in patients with UON

**Figure 3:**
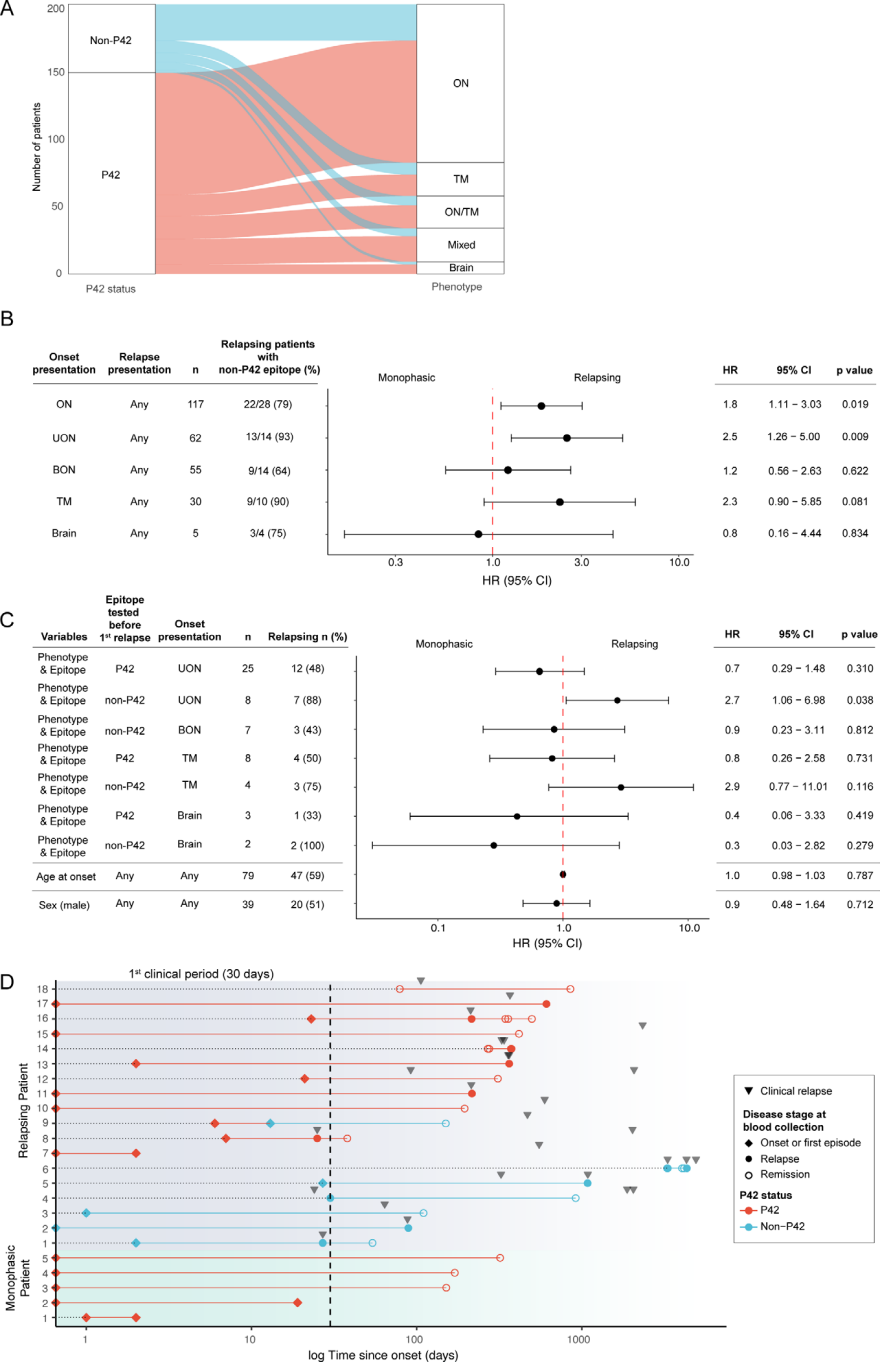
Non-P42 MOG-IgG was the strongest predictor of a relapsing course in patients with UON at onset. (A) Alluvial plots representing the distribution of MOG-IgG epitope status across MOGAD clinical phenotypes including ON, TM, ON/TM, mixed and brain. Patients with ON included UON and BON. (B) Univariate Cox proportional hazard model showing the risk of a relapsing course in patients with non-42 MOG-IgG within each phenotype category compared with the reference group (patients with P42 MOG-IgG within that phenotype category). (C) Multivariable Cox proportional hazard model showing the risk of a relapsing course in patients with UON, BON, TM and brain phenotypes at onset and whose non-P42 MOG-IgG were tested before the first relapse. The reference patients were patients with BON and non-P42 MOG-IgG. (D) Longitudinal serial samples from relapsing (top; purple background) and monophasic patients (bottom; green background) showing that MOG-IgG epitopes remain highly stable over time irrespective of treatment. Patient sera were assessed from the first sample to the last sample collected for a median duration of 126 days in relapsing patients and 160 days in monophasic patients. Basic information on treatment or lack thereof at time of blood collection were available for 10 patients and treatments included corticosteroids, rituximab, mycophenolate or untreated. Samples collected at onset or during the first clinical episode (within 30 days of the onset, shown by the dotted line) were defined as ‘sample collected at onset or the first episode’, and diamonds were used to represent these blood collections. Inverted triangles show clinical relapses. BON, bilateral optic neuritis; ON, optic neuritis; TM, transverse myelitis; UON, unilateral optic neuritis.

We next investigated whether associations between individual epitopes and disease course varied across MOGAD phenotypes. Both groups of patients whose serum MOG-IgG bound P42 and non-P42 epitopes displayed similar phenotypic profiles, with ON and TM being the most common phenotypes in both groups (figure 3A).

Since many MOGAD patients experience subsequent attacks that are phenotypically diverse, for example, ON then TM or BON then UON, patients were examined based on the phenotype at onset: ON, UON, BON, TM and brain followed by any clinical relapse (figure 3B). Of patients presenting with ON, those with non-P42 MOG-IgG exhibited 80% higher risk of a relapsing course compared with those with P42 MOG-IgG (HR 1.8; 95% CI 1.11 to 3.03; p=0.02; figure 3B). Patients with UON at onset and non-P42 MOG-IgG exhibited more than double the risk of a relapsing course compared with those with P42 MOG-IgG (HR 2.5;95% CI 1.26 to 5; p=0.01; figure 3B). In contrast, in patients with BON or a brain phenotype at onset, MOG-IgG binding to non-P42 epitope did not significantly increase the hazard of having a relapsing course compared with P42 epitope binding (p>0.5; figure 3B). Patients with TM and non-P42 MOG-IgG showed 2.3 higher hazard of a relapsing course than those with P42 MOG-IgG, although this did not reach significance (95% CI 0.90 to 5.85; p=0.08; figure 3B).

When patients having the same clinical phenotype throughout the disease course were analysed, non-P42 MOG-IgG was associated with a relapsing course among patients with ON, who had double the risk of experiencing a relapsing course than ON patients with P42 MOG-IgG (p=0.055, online supplemental figure 5). BON patients with non-P42 MOG-IgG did not show an increased risk of exhibiting a relapsing course compared with those with P42 MOG-IgG (online supplemental figure 5). However, UON patients with a non-P42 MOG-IgG almost exclusively exhibited a relapsing course (9/10, 90%) and had more than double the risk of a relapsing course compared with patients with UON and P42 MOG-IgG (HR 2.4; 95% CI 1.04 to 5.48; p=0.04; online supplemental figure 5). Notably, disease severity at onset varied between patients with UON and BON. Patients with BON had significantly higher VFSS scores at onset compared with patients with UON, and a similar trend was observed in EDSS scores (online supplemental figure 6A,B). Among patients with TM, 5/6 (83%) of patients with non-P42 MOG-IgG relapsed compared with those with P42 MOG-IgG (HR 2.8; 95% CI 0.81 to 9.80; p=0.1; online supplemental figure 5).

Multivariable Cox proportional hazard model on patients who had a sample collected before the first relapse revealed that compared with patients with BON and P42 MOG-IgG, those with onset UON and non-P42 MOG-IgG exhibited more than double the risk of exhibiting a relapsing course (HR 2.7; 95% CI 1.06 to 6.98; p=0.038; figure 3C). Relapse freedom in these patients lasted significantly shorter than those with a P42 MOG-IgG (p=0.0042, online supplemental figure 7). Other variables, such as age at onset and sex, did not associate with a relapsing course (figure 3C).

### Non-P42 MOG-IgG epitopes remained highly stable over time

Although MOG-IgG detection in serum at onset is recommended,^2^ the first sample may be collected during remission. To evaluate the stability of MOG-IgG epitopes over time, 57 sera were collected from 18 relapsing and 5 monophasic MOG-IgG-seropositive patients at disease onset or remission before first relapse, and subsequent serial samples collected at different time points throughout their disease course (figure 3D). Seventeen out of 18 (94%) relapsing patients and 5/5 monophasic patients had sera that bound to the same MOG-IgG epitope over time (figure 3D), with no change between onset or remission before first relapse, and subsequent collected sera, and irrespective of treatment or lack thereof (figure 3D).

### Non-P42 MOG-IgG combined with onset UON strongly predicted an MOGAD relapsing disease

**Figure 4:**
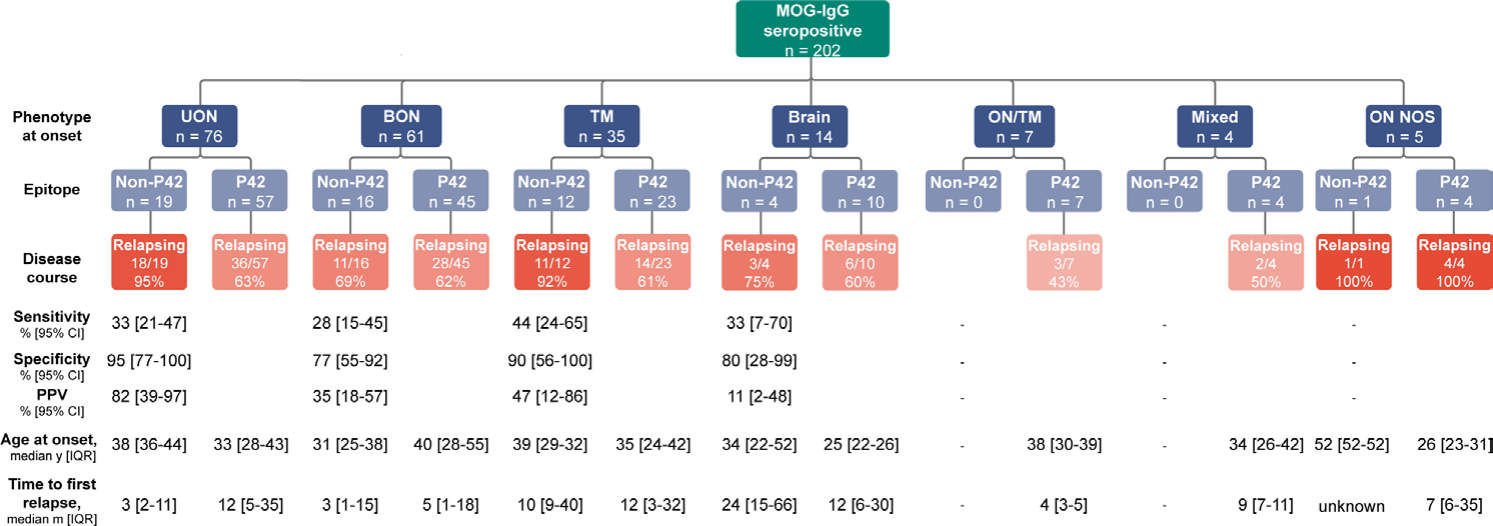
Distribution of non-P42 MOG-IgG in myelin oligodendrocyte glycoprotein antibody-associated disease (MOGAD) at onset. Patients with different MOGAD phenotypes at onset were stratified into their P42 epitope status and subsequently their disease course. Age at onset and time to first relapse are shown (median and IQR). There was no statistical difference between epitope groups for age at onset and time to first relapse. Sensitivity, specificity and PPV could not be determined in some groups due to the absence of patients with MOG-IgG non-P42 epitope in the ON/TM and Mixed groups, and the absence of patients with a monophasic course in the ON (NOS) group. BON, bilateral optic neuritis; NOS, not otherwise specified; PPV, positive predictive value, TM, transverse myelitis; UON, unilateral optic neuritis.

At onset, MOG-IgG-seropositive patients mainly presented with UON (n=76, 38%), BON (n=61, 30%), TM (n=35, 17%) or brain (n=14, 7%) (figure 4). A quarter of patients with UON and a third with TM had non-P42 MOG-IgG. Detection of the non-P42 epitope was associated with a relapsing course in 95% of UON and 92% of TM, but only in 69% and 75% of BON or brain patients, respectively. Specificity and positive predictive value were high in the UON group.^24^ Age at onset and time to first relapse were not different between groups, suggesting the prognosis utility of testing non-P42 MOG-IgG at onset.

## Discussion

This study provides definite evidence of the utility of non-P42 MOG-IgG epitopes as prognostic biomarkers for relapsing disease in MOGAD. The overall risk of a relapsing course in patients whose MOG-IgG recognised a non-P42 epitope was over three times higher than those who recognised the immunodominant P42 epitope. A seropositive non-P42 MOG-IgG test predicted a relapsing course in adult patients with UON with high specificity and positive predictive value. This association was particularly robust at disease onset, making MOG-IgG epitope detection a reliable predictor that can be tested before the occurrence of the first relapse.

Adults with MOGAD experience motor disability in 20%–40% of cases, and up to 20% experience lasting visual acuity impairments. Over 60% of patients with relapsing MOGAD had some residual deficit in one or more domains.^1 11 14^ Disability appears to be driven by acute clinical episodes,^14 32^ which was also evidenced here, where patients with relapsing disease exhibited significantly higher motor and visual disability at last review compared with monophasic patients. Therefore, the identification and appropriate treatment of relapsing patients is crucial to minimise disability. In the current study, there were no differences in motor and visual function scores at onset between monophasic and relapsing patients. This suggests the benefit of additional, non-clinical tools such as epitope testing to predict a relapsing course. This may also facilitate informed decision-making regarding therapeutic approaches including the early identification of patients to initiate maintenance immunotherapy, and the selection of patients for clinical trials designed for relapsing patients. Current treatment strategies are largely based on empirical data and clinical trials—which have only recently commenced for MOGAD (NCT05271409; NCT05063162)—are crucial for evidence-based management of the disease.

Our study significantly builds on our previous observation in which relapsing patients with ON were more likely to recognise an MOG-IgG epitope outside P42.^27^ Our current data, in a cohort more than double the size with extended follow-up of a minimum of 1 year, explore robust statistical modelling of relapse risk according to two MOG-IgG epitopes within different MOGAD clinical phenotypes and highlights the value of non-P42 MOG-IgG testing for clinical purposes. The association between a non-P42 epitope and a relapsing course was more than two times higher in patients with UON. Specificity and positive predictive value were high and comparable to those of the widely requested oligoclonal band in multiple sclerosis. Hence, the prognostic utility of a non-P42 epitope is highly applicable to adult MOGAD patients as UON is one of the most common presenting syndromes among adults.^2 11 22 31^ In contrast, a non-P42 epitope was not a predictor of a relapsing course among patients with BON although MOG-IgG prevalence is higher in BON.^2 33^ A pathophysiological reason for this divergence is not immediately apparent. One possible explanation for this divergence is that patients with BON, who in the present cohort exhibited higher EDSS and VFSS scores at onset compared with UON patients, may have undergone more aggressive treatment regimens due to the severity of their condition. Indeed, prior studies have demonstrated that steroid treatment for at least 1 month at onset has been associated with a monophasic disease course.^34^ While detailed therapeutic data were not available for the entirety of this cohort, this would be an important focus for future investigation.

We show here that non-P42 MOG-IgG is highly stable over time, similar to the P42 epitope.^27^ This enables the use of patient serum collected at onset or remission before a relapse for the prediction of a relapsing course. Although data were limited, epitope stability did not seem to be affected by patient treatment at sample collection, and a specific MOG-IgG epitope was not associated with acute immunotherapy during the first clinical episode or lack thereof. While non-P42 MOG-IgG can predict a short-term relapsing course, it cannot predict when a relapse occurs. Previous studies have found several non-IgG biomarkers of active disease compared with remission. These include sNfL,^35^ tau,^36^ TNFAIP3^37^ and a distinct CSF cytokine profile.^38 39^ A marker that exhibits dynamic levels based on disease activity and is scalable for use in diagnostics would be useful for predicting the timing of a relapse once patients at high risk of a relapsing course are identified with MOG-IgG epitope testing.

A biological explanation for the association between non-P42-targeting MOG-IgG and enhanced likelihood of relapsing disease in MOGAD has not yet been proposed. In Neuromyelitis optica syndrome disorder (NMOSD), AQP4-IgG with a H151/L154 epitope self-assembled into multimeric AQP4-IgG complexes resulting in efficient C1q binding and activation of the pathogenic complement cascade.^40^ This occurred despite low levels of bound IgG. In contrast, AQP4-IgG with a non-H151/L154 epitope required higher levels of IgG binding to trigger C1q binding and activation.^40^ Comparatively, it is possible that non-P42-targeting MOG-IgG can bind to MOG at low titers despite most MOG-IgG having low affinity,^27 41^ ultimately resulting in a relapse attack, whereas P42 epitope-binding MOG-IgG may require greater IgG levels to trigger a relapse.

A limitation of the current study is the lack of information concerning treatment regimen and potential confounding effect of treatment on relapse such as the type of treatment, delay in treatment administration and whether patients were under maintenance therapy. Reduced likelihood of relapses has been observed in patients on prednisolone and/or first-line immunosuppression,^14 22^ and thus the frequency of a relapsing course in the natural history of an untreated patient may have been underestimated as many patients were on active immunotherapy. The cohort included a higher percentage of relapsing MOGAD than previously reported in other cohorts,^11 31^ potentially due to an underrepresentation of monophasic patients whose follow-up time may be shorter. Furthermore, a non-P42 epitope was present in 26% of the MOGAD cohort, rendering the prognostication valid only for a quarter of the MOGAD population. In contrast, P42-binding MOG-IgG-seropositive patients constituted 74% of the cohort but did not display a preference for either disease course, thereby making this epitope unsuitable for predicting their clinical trajectory. Although the precise binding site of MOG-IgG with a non-P42 and non-H103/S104 epitope and potential additional binding sites outside P42 and H103/S104 for P42-binding sera remains unknown, prospective studies are needed to investigate the risk of relapsing disease among these patients. Further elucidation of the non-P42 epitope could include investigations on the binding to S42, binding to altered structures of MOG induced by the P42S mutation, binding to altered structures of MOG not centred on residue 42, or a combination thereof.

Strengths of this study include high feasibility and the ease of implementation of this prognostic biomarker in serum which is the recommended biospecimen for MOG-IgG testing. Although the lack of binding to H103/S104 together with the lack of binding to P42 showed high risk of a relapsing course, our data demonstrate that specificity and high positive predictive value can be achieved with testing P42 MOG-IgG alone, rather than both P42 and H103/S104, strongly increasing the feasibility of the test for clinical purposes. Although several methodologies are currently reported for the live assay,^2 25 42^ the determination of the non-P42 epitope status can be conducted with various secondary antibody isotypes and without a control cohort after ROC analysis, ensuring high applicability of this diagnostic test in MOG-IgG testing centres globally.

In summary, the identification of non-P42 MOG-IgG presents a significant breakthrough in prognosticating a relapsing disease course in adult patients with MOGAD, especially with UON. To our knowledge, this is the first and only test available that enables early identification of patients at risk of experiencing a relapsing course and may be a valuable tool in the effort to minimise relapse rates, ensure appropriate selection of patients for immunotherapy and mitigate disability in MOGAD.

## Data Availability

Data are available on reasonable request. Deidentified data from the Australasian MOGAD Study Group are available by request through the senior corresponding author. All requests will be reviewed by study leadership, and if approved, data will be transferred subject to an institutional data use agreement. The epitope data are available on reasonable request made to the senior corresponding author. All requests should agree with the Australian legislation on the general data protection regulation and decisions by the Human Ethics Committee of Australia. If approved, data will be transfered subject to a material transfer agreement.
