# Supplementary material for: The MOG antibody non-P42 epitope is predictive of a relapsing course in MOG antibody-associated disease
Source: J Neurol Neurosurg Psychiatry. 2024 Jan 30;95(6):544–53. doi: 10.1136/jnnp-2023-332851 (PMC11103329; doi:10.1136/jnnp-2023-332851)
Supplement: Supplementary data [file jnnp-2023-332851supp001.pdf]

Supplementary File

Liyanage *et al.* The MOG antibody non-P42 epitope is predictive of a relapsing course in MOG antibody-associated disease

Supplementary Methods

MOG-IgG Epitope Testing by flow cytometry live cell-based assay

Serum IgG binding to full-length wild-type human MOG α1 isoform (MOG WT), MOG P42S and MOG H103A/S104E, and an empty mCherry vector control (CTL) were assessed in the research-based flow cytometry live cell-based assay (CBA) as previously described. [1-3] The MOG P42S mutant contained a mutation at position 42, where the Proline substituted for Serine. [3, 4] The MOG H103A/S104E mutant consisted of full-length human MOG with the histidine and serine at positions 103 and 104 substituted with alanine (103A) and glutamic acid (104E).[4] Stable cell lines were generated by lentiviral transduction in human embryonic kidney (HEK293) cells as previously described.[5-7] MOG WT, MOG P42S and MOG H103A/S104E cell surface expression was controlled in each experiment, were similarly high as described.[3] The P42 and H103/S104 MOG-IgG epitope statuses were calculated originally using a control cohort as previously described.[3] Briefly, MOG-IgG binding to MOG WT, MOG P42S and MOG H103A/S104E was determined by the delta median fluorescence intensity (ΔMFI): MOG WT, or MOG P42S, or MOG H103A/S104E ΔMFI = MOG MFI – CTL MFI. Epitope status of each patient serum was first calculated using control sera to establish a control reference range by calculating the 3SD above and below the control P42 MOG-IgG or H103/S104 MOG-IgG mean. MOG-IgG were assigned as P42 or H103/S104 MOG-IgG (above mean + 3SD) and non-P42 or non-H103/S104 MOG-IgG (below mean + 3SD). Three independent experiments were repeated, and reported samples remained in the same category in at least two of three independent experiments with low inter-assay variability. Then, to remove the need for a control cohort and facilitate global adoption of the method by diagnostic providers, a receiver-operating-characteristic (ROC) analysis was used to determine optimal thresholds for P42 and MOG H103/S104 MOG-IgG binding (online supplemental figure 1A-D). These thresholds were utilised for calculation of epitopes in this study. Flow cytometry data was acquired on the LSRII flow cytometer (BD Biosciences) and analysed using FlowJo v10 (TreeStar) software and Microsoft Excel.

Supplementary Tables

Supplemental Table 1. Severity characteristics of MOGAD patients stratified by P42 epitope serostatus.

	**EDSS median [IQR], n[a]**				**VFSS median [IQR], n[a]**			
**MOG-IgG Epitope**	**Onset**	**Last review**	**P value**	**ΔEDSS**	**Onset**	**Last review**	**P value**	**ΔVFSS**
P42 and H103/S104	3.0 [2.0-3.5], 45	1.0 [0-2.0], 45	<0.001	2.0	3.0 [2.0-3.50], 37	1.0 [0-2.0], 37	0.001	2.0
P42 and non-H103/S104	3.0 [2.0-4.0], 41	1.0 [0-2.0], 41	<0.001	2.0	3.0 [2.0-4.0], 37	1.0 [0-2.0], 37	<0.001	2.0
Non-P42 and H103/S104	3.0 [2.0-3.8], 19	1.0 [0.5-3.0], 19	0.006	2.0	3.0 [2.8-3.6], 17	2.0 [1.0-3.0], 17	0.004	1.0
Non-P42 and	3.0 [3.0-	2.0 [1.0-	0.021	1.0	3.0 [3.0-	2.0 [1.0-	0.1	1.0

Liyanage G, *et al. J Neurol Neurosurg Psychiatry* 2024; 95:544–553. doi: 10.1136/jnnp-2023-332851

non-H103/S104	4.0], 9	3.0], 9			4.0], 9	3.0], 9		

Abbreviations: EDSS, Expanded Disability Status Scale; VFSS, Visual Functional Systems Score.
[a]number of patients for whom paired data was available.

Supplemental Table 2. Demographic, clinical, and MOG-IgG characteristics of relapsing MOGAD patients stratified by P42 epitope serostatus.

Variable	Total	P42	Non-P42	p value[a]
n	137	93	44	
Age, median y [IQR]	40.53 [31.60-50.75]	39.83 [31.33-51.98]	40.94 [34.41-49.52]	0.989
Sex – male, n (%)	54 (39.4)	36 (38.7)	18 (40.9)	0.853
Follow-up, median y [IQR]	5.33 [2.47-9.60]	5.61 [2.50-9.86]	5.05 [2.28-9.37]	0.365
Time to first relapse, median m [IQR], n[b]	7.03 [3.00-24.63], 113	7.00 [3.08-22.82], 79	8.23 [2.29-26.31], 34	0.684
ARR, median [IQR], n[b]	0.38 [0.19-0.57], 86	0.38 [0.18-0.55], 61	0.38 [0.25-0.62], 25	0.43
Phenotype at onset, n (%)				0.736
UON	54 (39.4)	36 (38.7)	18 (40.9)	
BON	39 (28.5)	28 (30.1)	11 (25.0)	
ON (Unspecified)	5 (3.6)	4 (4.3)	1 (2.3)	
ON/TM	3 (2.2)	3 (3.2)	0 (0.0)	
TM	25 (18.2)	14 (15.1)	11 (25.0)	
Brain	9 (6.6)	6 (6.5)	3 (6.8)	
Mixed	2 (1.5)	2 (2.2)	0 (0.0)	
Phenotype over whole disease course, n (%)				0.602
UON	40 (29.2)	26 (28.0)	14 (31.8)	
BON	9 (6.6)	7 (7.5)	2 (4.5)	
ON (Unspecified)	2 (1.5)	1 (1.1)	1 (2.3)	
ON (mixed)	24 (17.5)	19 (20.4)	5 (11.4)	
ON/TM	19 (13.9)	12 (12.9)	7 (15.9)	
TM	15 (10.9)	8 (8.6)	7 (15.9)	
Brain	4 (2.9)	2 (2.2)	2 (4.5)	
Mixed	24 (17.5)	18 (19.4)	6 (13.6)	
EDSS, median [IQR]				
Onset, n[b]	3.00 [2.00-4.00], 78	3.00 [2.00-4.00], 54	3.00 [2.75-4.00], 24	0.395

Liyanage G, *et al. J Neurol Neurosurg Psychiatry* 2024; 95:544–553. doi: 10.1136/jnnp-2023-332851

Last review, n[b]	1.50 [0.00-3.00], 104	1.50 [0.00-3.00], 73	1.00 [1.00-2.75], 31	0.983
VFSS, median [IQR]				
Onset, n[b]	3.00 [0.50-5.00], 71	3.00 [0.75-5.00], 48	3.00 [0.50-5.00], 23	0.955
Last review, n[b]	1.00 [0.00-2.00], 94	1.00 [0.00-2.00], 66	0.50 [0.00-2.00], 28	0.572
Time from onset to first sample, median m [IQR]	9.81 [0.70, 78.58]	6.68 [0.48, 82.83]	11.34 [0.87, 74.32]	0.726
MOG-IgG titer (clear positive (%)[c]	39 (95.1)	29 (100.0)	10 (83.3)	0.080

Abbreviations: AAR, annualised rate of relapse; EDSS, Expanded Disability Status Scale; VFSS, Visual Functional Systems Score.

[a]P values are computed based on comparisons between patients with P42 MOG-IgG and patients with non-P42 MOG-IgG.

[b]number of patients with ON, TM, Brain, and Mixed phenotypes for whom the data was available.

[c]MOG-IgG titer was calculated for patient sera collected at onset.

Supplementary figures

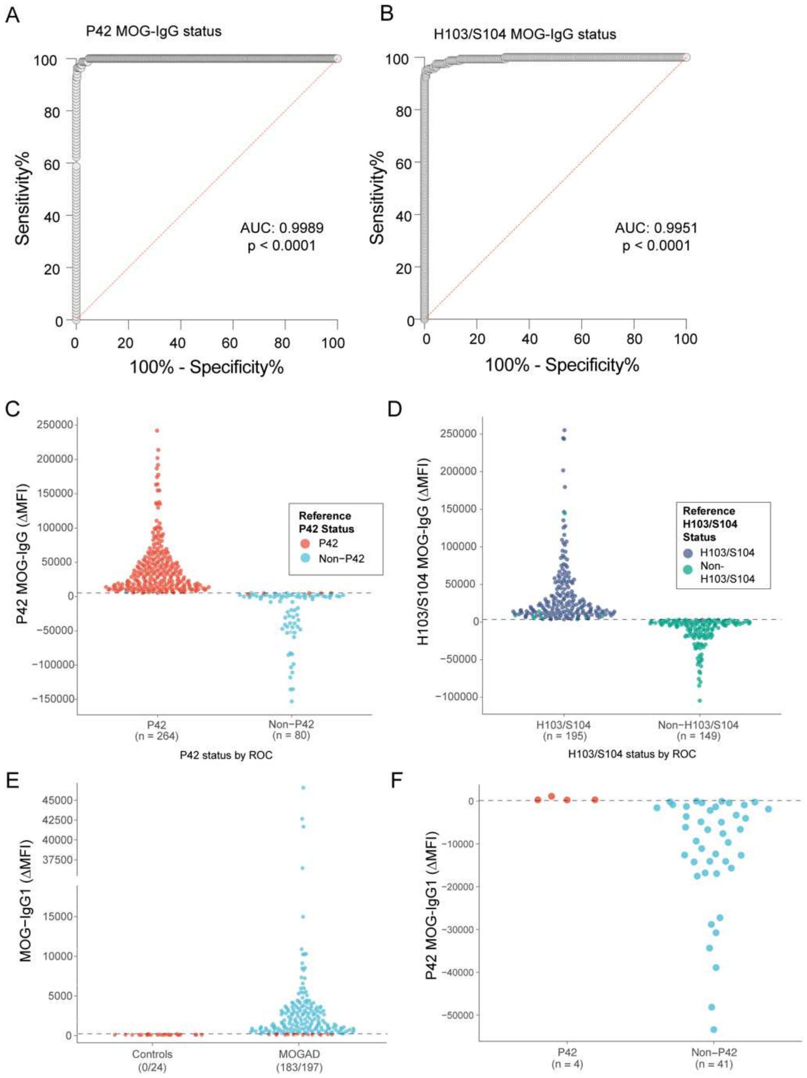

Supplemental figure 1. Applicability of MOG-IgG epitope status determination to other testing centres. Receiver operating characteristic (ROC) curves were generated for P42 MOG-IgG (A) and H103/S104 MOG-IgG (B) epitope status determination to establish optimal binding thresholds that result in high sensitivity and specificity without the use of a control cohort (patients from general medical and non-inflammatory neurological disorder patients, n=24). 344 sera from MOGAD patients (143 without disease course data) were used. P42 and H103/S104 epitope status was concordant in 338/344 (98%) and 334/344 (97%) patients, respectively, compared to when the 24 controls were used. MOG-IgG epitopes, either P42 and non-P42, or H103/S104 and non-H103/S104, could be clearly discriminated (AUC=0.9989, p< 0.0001, and AUC=0.0.9951, p<0.0001). P42 epitope (C) and H103/S104 epitope (D) as determined by ROC-generated optimal thresholds were compared

to the reference epitope status for each patient as determined using a control cohort, and results were highly concordant between analyses methods. (E) The vast majority of patient sera positive for MOG-IgG using a secondary anti-human IgG (H+L) antibody were also positive for MOG-IgG1 using a secondary anti-human IgG1 antibody in a flow cytometry live cell-based assay. (F) Epitope status was determined using a secondary anti-human IgG1 antibody in 45 patient sera that displayed a non-P42 MOG-IgG when using secondary anti-human IgG (H+L) antibody. Most patients (41/45) displayed the same non-P42 epitope when tested with IgG1. Of these 41 patients, 34 (83%) exhibited a relapsing course. Abbreviations: AUC, area under the curve.

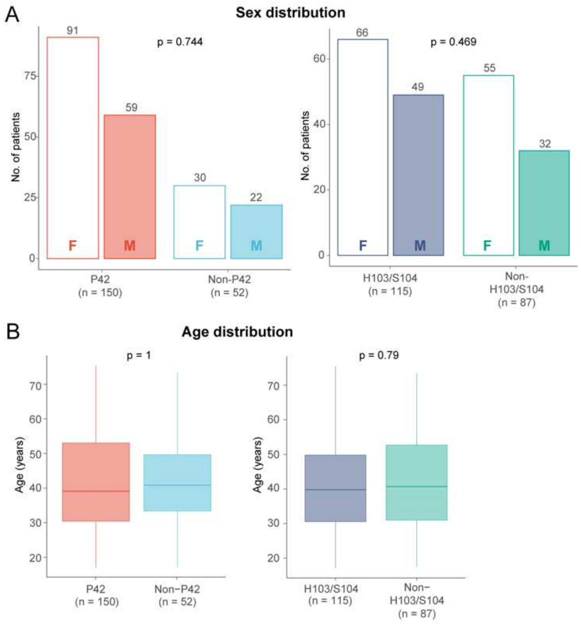

Supplemental figure 2. Patient sex and age distribution across MOG-IgG epitopes.
Bar plots showing the number of male and female patients within the MOGAD cohort (n=202) stratified by MOG-IgG P42 (left) and H103/S104 (right) epitope status (A). Box-and-whisker plots showing the distribution of patient ages at the date of sera collection stratified by MOG-IgG P42 (left) and H103/S104 (right) epitope status (B). The time from onset to first sample collection was not different between P42 (median 2.2 months, IQR 0.17 - 40.0) and non-P42 MOG-IgG epitope groups (median 5.7 months, IQR 0.16 – 59.9), p = 0.278. Abbreviations: F, female; M, male.

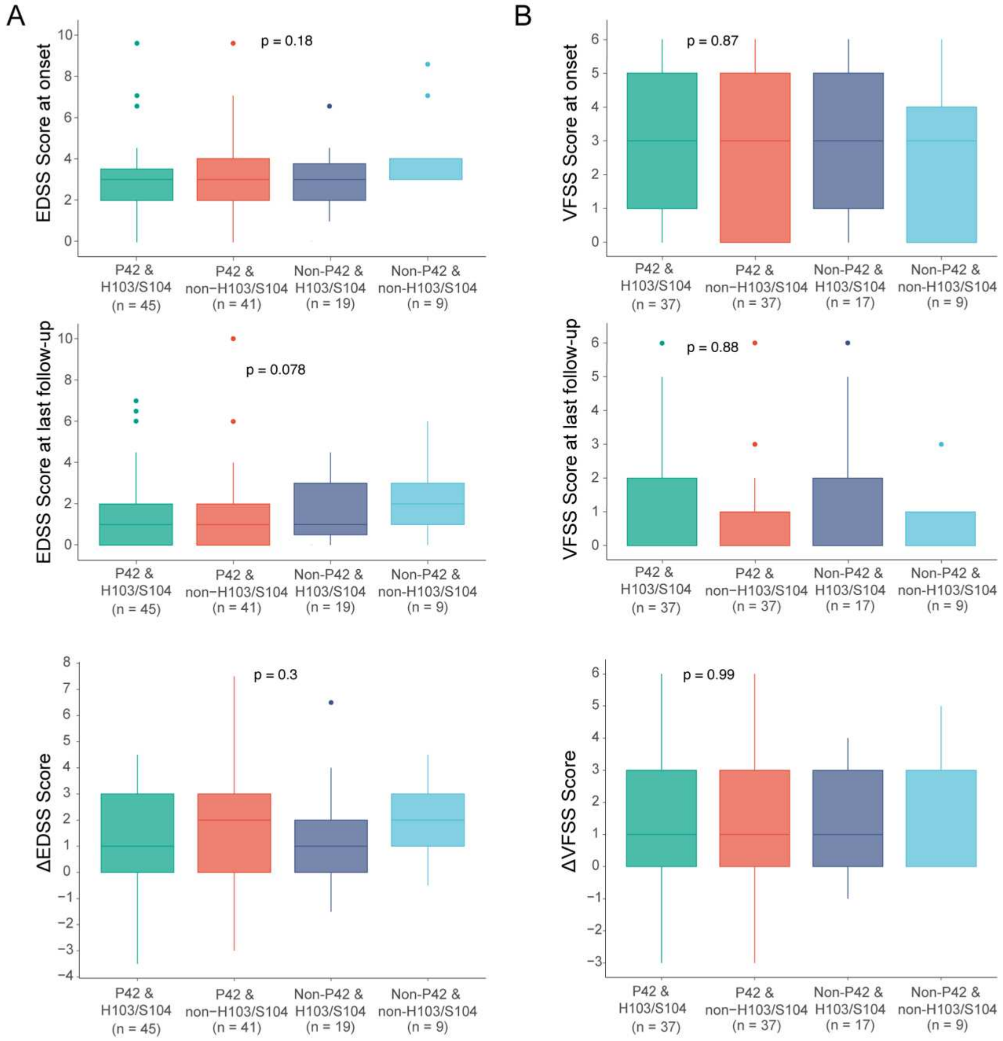

Supplemental figure 3. MOG-IgG epitopes were not associated with severity in MOGAD patients. EDSS (A) and VFSS (B) scores at disease onset (top graphs), last follow-up date (middle graphs), and difference between onset and last follow up date (delta, bottom graphs) were compared between patients who presented with the four combinations of the MOG-IgG epitopes. There was no significant association between severity scores (delta) and epitope groups (Kruskal-Wallis test). Abbreviations: EDSS, Expanded Disability Status Scale; VFSS, Visual Functional Systems Score.

Liyanage G, *et al. J Neurol Neurosurg Psychiatry* 2024; 95:544–553. doi: 10.1136/jnnp-2023-332851

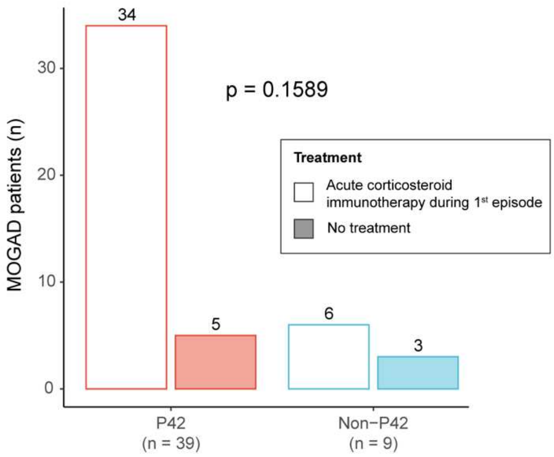

Supplemental figure 4. Treatment during the first clinical episode in patients with P42 and non-P42 MOG-IgG. Administration of acute corticosteroid immunotherapy or lack thereof were compared between patients with P42 MOG-IgG (n=39 total; Treated: n=15 BON, n=8 UON, n=4 ON/TM, n=4 TM, n=2 ON NOS, and n=1 Mixed; Untreated: n=4 UON, and n=1 ON NOS) and non-P42 MOG-IgG (n=9 total; Treated: n=3 BON, n=2 UON, n=1 TM; Untreated: n=1 BON, n=1 UON, and n=1 TM). Acute corticosteroid immunotherapy included intravenous pulsed methylprednisolone and oral corticosteroid taper, or intravenous pulsed methylprednisolone only. There was no significant association between acute corticosteroid immunotherapy during the first clinical episode and the two epitope groups (Chi-square and Fisher's exact Test). Abbreviations: T, treated; U, untreated.

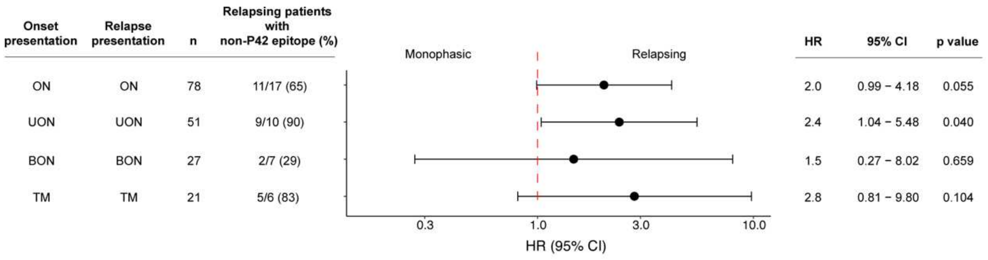

Supplemental figure 5. Non-P42 MOG-IgG was the strongest predictor of a relapsing course in patients with UON at onset. Univariate Cox Proportional Hazard Model showing the risk of a relapsing course in patients with non-42 MOG-IgG and the same clinical phenotype throughout the course of their MOGAD disease. Reference groups were patients with P42 MOG-IgG with the same clinical phenotype throughout MOGAD course. Abbreviations: BON, bilateral optic neuritis; ON, optic neuritis; UON, unilateral optic neuritis; TM, transverse myelitis.

Liyanage G, *et al. J Neurol Neurosurg Psychiatry* 2024; 95:544–553. doi: 10.1136/jnnp-2023-332851

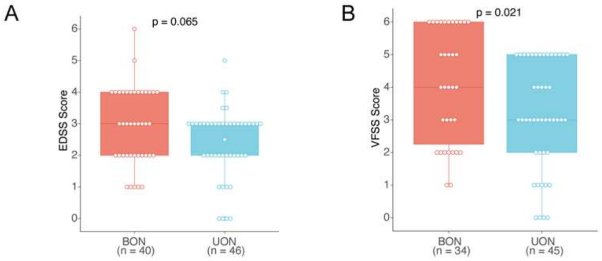

Supplemental figure 6. Patients with BON had higher disease severity scores at onset. Measures of disease severity according to EDSS (A) and VFSS scores (B) were compared between patients who presented with BON at onset and those with UON at onset. Among patients with a known VFSS score, follow-up time (BON: median 3.3 years (IQR 1.7-5.1), UON: median 3.5 years (IQR 2.2-6.2)), the proportion of relapsing patients (BON: 68%, UON: 67%), and their time to first relapse (BON (n=22): median 4.1 months (IQR 1.4 – 13.8); UON (n=28): median 7.0 months (IQR 3.3 – 15.0); p=0.13) were not different. Abbreviations: BON, bilateral optic neuritis; EDSS, Expanded Disability Status Scale; UON, unilateral optic neuritis; VFSS, Visual Functional Systems Scores.

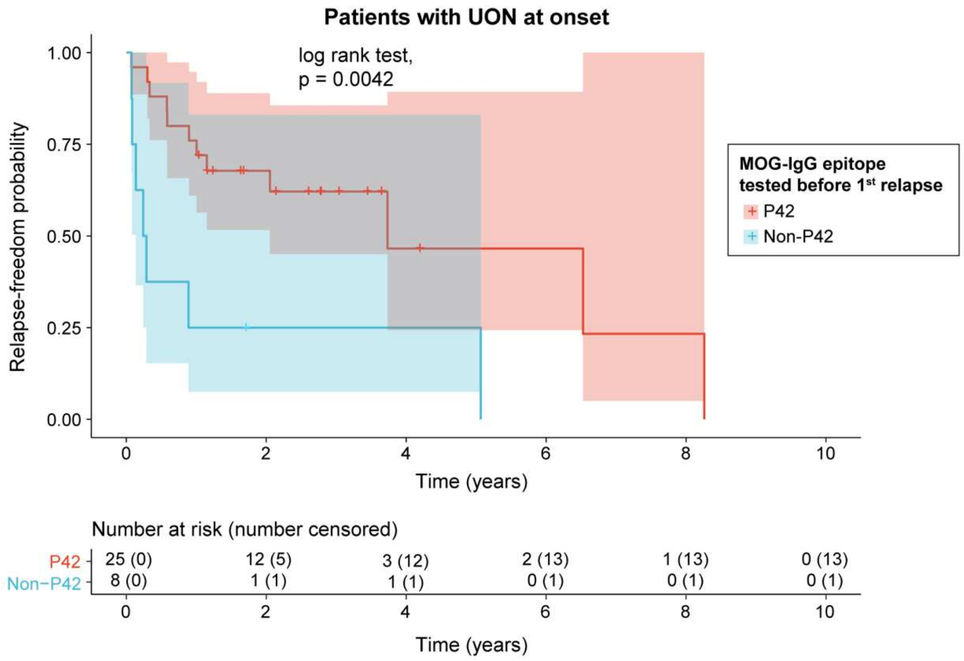

Supplemental figure 7. Relapse-freedom in patients with UON at onset and non-P42 MOG-IgG detected prior the first relapse was significantly shorter than those with P42 MOG-IgG. Kaplan-Meier curve showing the relapse-freedom period of patients presenting with UON at onset with P42 MOG-IgG compared to a non-P42 MOG-IgG (number of censored observations and number at risk are shown below the curve). 95% CI are shown in

shaded areas on both sides of the survival curves. Abbreviations: UON, unilateral optic neuritis.

Supplementary References

1. Lopez JA, Houston SD, Tea F, et al. Validation of a Flow Cytometry Live Cell-Based Assay to Detect Myelin Oligodendrocyte Glycoprotein Antibodies for Clinical Diagnostics. J Appl Lab Med 2022;7:12-25.
2. Reindl M, Schanda K, Woodhall M, et al. International multicenter examination of MOG antibody assays. Neurology - Neuroimmunology Neuroinflammation 2020;7:e674.
3. Tea F, Lopez JA, Ramanathan S, et al. Characterization of the human myelin oligodendrocyte glycoprotein antibody response in demyelination. Acta neuropathologica communications 2019;7.
4. Mayer MC, Breithaupt C, Reindl M, et al. Distinction and Temporal Stability of Conformational Epitopes on Myelin Oligodendrocyte Glycoprotein Recognized by Patients with Different Inflammatory Central Nervous System Diseases. The Journal of Immunology 2013;191:3594-3604.
5. Dale RC, Tantsis EM, Merheb V, et al. Antibodies to MOG have a demyelination phenotype and affect oligodendrocyte cytoskeleton. Neurology - Neuroimmunology Neuroinflammation 2014;1:e12.
6. Menge T, Lalive PH, von Büdingen HC, Genain CP. Conformational epitopes of myelin oligodendrocyte glycoprotein are targets of potentially pathogenic antibody responses in multiple sclerosis. J Neuroinflammation 2011;8:161.
7. Ramanathan S, Reddel SW, Henderson A, et al. Antibodies to myelin oligodendrocyte glycoprotein in bilateral and recurrent optic neuritis. Neurology - Neuroimmunology Neuroinflammation 2014;1:e40.
